# Oropharyngeal Rhabdomyosarcoma with cranial nerve paralysis in a limited resource setting: a case report and review of literature

**DOI:** 10.11604/pamj.2019.34.51.20255

**Published:** 2019-09-25

**Authors:** Askia Alfazaz, Ibrahim Assoumane, Ousseini Adakal, Harissou Adamou, Ibrahim Amadou Magagi, Ibrahim Baaré

**Affiliations:** 1 ENT Department, CHR of Maradi, Maradi, Niger Republic; 2 Neurosurgery Department, Reference Hospital Maradi, Maradi, Niger Republic; 3 Department of General Surgery, Faculty of Health Sciences, University of Maradi, Maradi, Niger; 4 Department of General Surgery, Faculty of Health Sciences, University of Zinder, National Hospital, Zinder, Niger; 5 Department of Ophthalmology, Maradi Reference Center, Maradi, Niger Republic

**Keywords:** Rhabdomyosarcoma, child, oropharyngeal, recurrence

## Abstract

Rhabdomyosarcoma (RMS), a tumor of skeletal muscle origin, is the most common soft tissue sarcoma encountered in childhood and adolescence; it is primarily found in the head and neck region, it is relatively uncommon tumors of the oral cavity. Clinical signs depend on the exact location of the lesion in the oral cavity and its development. Authors reported the case of a 14-year-old patient who presented an oropharyngeal mass causing voice dysfunction, after two surgical operation the patient experimented two 2 recurrences of the lesion. The histopathological examination objectifies an oropharyngeal rhabdomyosarcoma. Immediate postoperative outcome was uneventful with improvement in the voice dysfunction and dysphagia one month after surgery. Complementary treatment (chemotherapy and radiotherapy) was not available and accessible to the patient. Twenty months (20) after surgery, the examination found a recurrence of the tumor with pulmonary metastases and neurological complications. Oropharyngeal rhabdomyosarcomas are rare. Their interest lies in the fact that they often affect children and adolescents. The prognosis remains unfavorable in our context, even for cases accessible to surgery since complementary treatment with chemotherapy and / or radiotherapy does not exist. The prognosis depends on tumor size, location, staging, age of patients and especially the quality of the management.

## Introduction

Rhabdomyosarcoma (RMS) constitutes a heterogeneous and aggressive malignant soft tissue neoplasm arises from undifferentiated mesodermal tissue [1-4]. RMS is primarily in striated muscle but can originate in the soft tissue that does not normally contain striated muscle [1, 3, 5]. RMS is the most common soft tissue sarcoma among children and adolescents [1, 4, 6]. This tumor accounts for 5% to 10% of all malignancies in childhood tumors [1, 5-7]. All parts of the body can be affected by RMS, but the most affected sites are the head and neck region in 40%, the genitourinary tract in 23%, the extremities in 20% and the other parts in 22% [3, 6, 8]. RMS located at the level of the head and neck can occur in the paramenigeal areas such as the orbit, ear, nasal cavity, sinus, nasopharynx and infratemporal fossa. Non-meningeal locations include the scalp, parotid gland, oral cavity, pharynx, thyroid and parathyroid [8-10]. The oropharyngeal cavity is even more rarely reported in the literature [1, 5, 9, 10]. The treatment of RMS must be multidisciplinary and depends on the site. Complete initial resection of the tumor with healthy margins is ideal [5-8]. However, chemotherapy (neo-adjuvant and/or adjuvant) and radiotherapy are used in addition to surgery or as therapeutic alternatives for inoperable cases [2, 10, 11]. In our context, the delayed diagnosis, and the lack of the technical platform are aggravating factors of this cancer which prognosis is already bad. Here we report the management of oropharyngeal rhabdomyosarcoma in a 14-year-old male patient at Maradi regional hospital (CHR).

## Patient and observation

**Figure 1:**
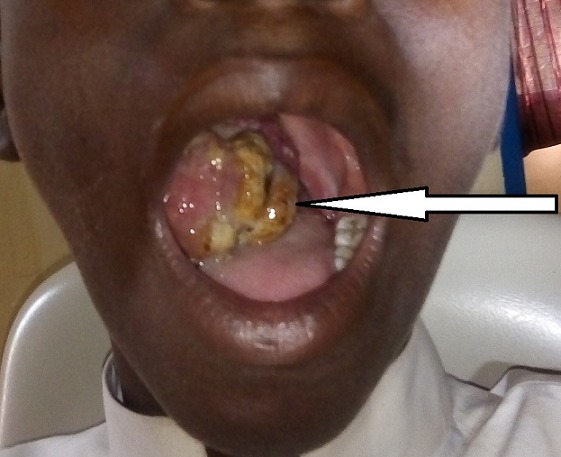
Aspect of the oropharyngeal tumor on clinical examination

A 14-year-old patient, from the rural area, with precarious socio-economic status, consulted at the Ear Nose Throat (ENT) department of Maradi CHR for an oropharyngeal mass evolving for several months. The last three months, the mass has increased in volume significantly. The patient had functional signs such as dysphonia, dysphagia and pain. At admission, the patient had a good general status. The conjunctiva and mucous membranes were normal-colored. At the opening of the mouth, the examination of the oropharyngeal cavity, noted an indolent mass, nodular, bloody and located on the right side of the oral cavity. The major axis of the mass measured approximately 5 to 6 cm (Figure 1). The biological investigations were without anomalies. Standard chests X-ray, abdominal and cervical ultrasound were normal. Computed tomography was not available in our hospital and the parents of the patients did not have the financial resources to evacuate their child to the capital Niamey which is about 600 miles away. The diagnosis of oropharyngeal tumor was made and the surgical indication of a biopsy excision was made. The child underwent general anesthesia with orotracheal intubation. The installation was done in order to obtain the extension of the neck and the head. After opening the mouth, the lesion was exposed, gross macroscopic resection of the tumor was performed. The specimen was sent for histopathological examination that revealed a malignant tumor proliferation with diffuse disposition, the cells had an oval nucleus and eosinophilic cytoplasm, as rhabdomyoblastic. The tumor cells have a perivascular disposition and there is a lot of mitosis. In conclusion, the diagnosis of an embryonal rhabdomyosarcoma botryoid variant was retained.

**Figure 2:**
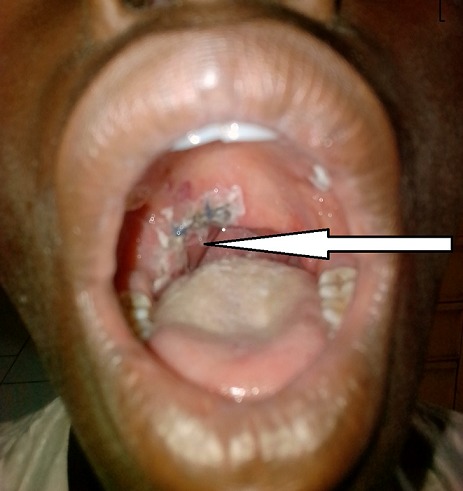
Postoperative appearance after tumor reduction surgery

**Figure 3:**
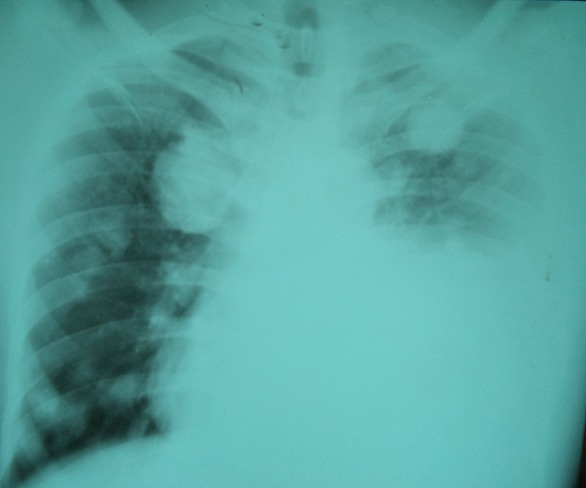
Chest X-ray showing metastasis and pleurisy on the left

Immediate postoperative outcome was uneventful with improvement in the voice dysfunction and dysphagia one month after surgery. The patient was referred to Niamey at the oncology department for adjuvant chemotherapy, but the parents reaffirm that they could not afford to bring their son to Niamey. After a return visit at 3 and 6 months at the CHR of Maradi, the patient was lost sight of. He returned 14 months after surgery with an oropharyngeal tumor recurrence. In addition to dysphagia and dysphonia, the patient had facial palsy ipsilateral to the tumor. The biological assessment noted anemia at 10 g/dl. The ultrasound showed images in favor of extending the tumor to the parotid. A whole blood transfusion was done to correct the anemia. A second tumor reduction surgery was performed (Figure 2). The immediate result was marked by an improvement in dysphonia and dysphagia. Six months after the second operation the patient was readmitted with a poor general condition, dysphonia, severe dyspnea, facial paralysis. He also presented the nine cranial nerve paralysis, taste dysfunction and tongue deficit. The examination found a recurrence of the tumor with a larger volume. Pulmonary radiography noted images of balloons released and left pleural effusion, reflecting the presence of pulmonary and lymph nodes metastases (Figure 3). After 7 days of symptomatic treatment, the patient was discharged at the request of the parents.

## Discussion

This study has limitations. Indeed, we perform the surgery in a context of limited resources. On the one hand, the patient comes from disadvantaged rural areas, consulted late and does not have sufficient financial resources to cope with the cost of care. On the other hand, our hospital does not have multidisciplinary services that allow a holistic treatment of patients. Our patient with oropharyngeal RMS did not receive any additional treatment (chemotherapy and radiotherapy) for the surgery. However, the surgical management of this RMS had improved the quality of life of our patient and the results of this experiment deserve to be discussed with the scientific literature. The first case of rhabdomyosarcoma was published in 1854 by Weber; it was probably a botryoid RMS of the tongue [4, 10, 11]. RMS constitute a heterogeneous and aggressive malignant soft tissue neoplasm arise from undifferentiated mesodermal tissue. It accounts for 3-4% of all cancers that develop during childhood [1-3, 7]. More than 50% of RMS occurs in the first decade of life [3]. Nearly 40% of cases are located in the region of the head and neck and the majority of cases of RMS are reported between 2 and 7 years [4-7, 9, 10]. Our 14-year-old patient was not in the high-risk category. For many authors the highest incidence of RMS is in the children aged 1-4 years, lower rate in children aged 10-14 aged and lowest rates 15-19 years of age [5, 7, 9]. The reported case is located in oropharyngeal region, which is not very common as assumed many authors [5, 7-10]. The most common site is the head and neck (parameningeal and orbit), followed by genitourinary tract. Less commonly involved sites of head and neck regions are the nasal cavity and nasopharynx, ears, paranasal sinuses, soft tissue of the face and neck [11, 12].

The histologic classification of rhabdomyosarcoma remains very controversial, as several distinct morphological variants have been reported [3, 4]. Horn and Enterline [13] were the first to propose the subdivision of RMS into 4 types botryoid, embryonal, alveolar and pleomorphic. Formerly, pathologic classification divided RMS into two main types embryonal and alveolar [1, 6]. The embryonal type presents the subtypes including spindle cell and botryoid [1, 6, 11, 12]. However, the current World Health Organization (WHO) classification [14], is closer to that proposed by Horn and Enterline [13]; it groups the RMS into four histologic groups: Type 1: embryonal rhabdomyosarcoma: A) botryoides variant; B) anaplastic variant. Type 2: alveolar rhabdomyosarcoma: A) solid variant; B) anaplastic variant. Type 3: pleomorphic rhabdomyosarcoma. Type 4: spindle cell/sclerosing rhabdomyosarcoma. It should be noted that pleomorphic RMS is most often found in adults, but rare in children [14]. The histolopathological type of our patient was embryonal rabdomyosarcoma botryoid variant. The diagnosis of RMS should be based not only on the histopathological aspect, but also on the immunohistochemical and molecular profiles of these tumors. These exams are not available in many developing countries. Molecular diagnosis not only confirms RMS but also monitors residual disease. It allows especially targeted treatment [1]. The treatment of RMS involves a multimodal approach. Treatment should be undertaken in oncology centers with multidisciplinary teams [7, 12, 15]. The treatment combines surgery, chemotherapy and adjuvants and/or neoadjuvant radiotherapy according to available expertise [3, 6, 7, 15]. Initial surgery can be considered in patients with low risk localized embryonal non-orbital non-parameningeal RMS [3, 7].

The cytotoxic actions of chemotherapeutic agents are not tumor-specific and are not effective in advanced and metastatic RMS [3, 6, 8, 10]. In our case, surgical resection was the only way available in our environment. Inevitably, recurrence occurred 16 months after surgery. Chemo-Radiotherapy is not available; let alone the targeted therapy. However, surgical resection had improved the quality of life of our patient. The usual regimens used in chemotherapy are: carboplatin/etoposide, ifosfamide/carboplatin/etoposide, vincristine/irinotecan, cyclophosphamide/topotecma, vinorelbine/cyclophosphamide [3, 10]. Radiotherapy chemotherapy improves the prognosis. Poorly treated tumors develop infiltrating and increase the risk of recurrence [8]. The overall survival of patients with RMS has improved considerably over the past two decades because of diagnostic and therapeutic progress [6-8]. The five-year survival rate of SMR has improved, from less than 10% before the 1960s to 65% today [10]. This survival at 5 can reach 85% for low-grade localized RMS [6]. This rate decreases considerably with age, height and advanced stages at the time of diagnosis of RMS [6], but remember that RMS is an aggressive tumor with rapid growth in children [11]. Nearly 25% of cases of RMS are metastatic at presentation [3, 6-8]. RMS are associated with high rates of recurrence metastases through the blood and/or lymphatic routes [10]. The most common site of metastasis was bone, followed by a lung and lymphadenopathy [7]. Twenty months after the 1^st^ surgical resection, our patient presented with pulmonary and lymph nodes metastases. For Kim *et al*. [7], the median time to metastasis of RMS was 14.0 months (1-85 months). Patients with smaller tumors (≤5 cm) have better survival. The volume of the tumor and its maximum diameter are associated with the outcomes [3]. At the first diagnosis, the tumor diameter for our patient was between 5 and 6 cm. Our patient's evolution was marked by recurrences, to which were added neurological complications of pulmonary metastases.

## Conclusion

The oropharyngeal rhabdomyosarcomas are rare. Their interest lies in the fact that they often affect children and adolescents. The prognosis remains unfavorable in our context, even for cases accessible to surgery since complementary treatment with chemotherapy and/or radiotherapy does not exist. The improvement of the technical platform for earlier diagnosis and multidisciplinary management is necessary to meet the experts' recommendations on the treatment (adjuvant and/neoadjuvant therapy after radical surgical excision) and follow-up of RMS.

## Competing interests

The authors declare no competing interests.
